# Animal and organoid models to elucidate the anti-fibrotic effect of steroid on biliary atresia

**DOI:** 10.1007/s00383-024-05798-7

**Published:** 2024-08-05

**Authors:** Fangran Liu, Vincent Chi Hang Lui, Zhongluan Wu, Paul David Blakeley, Clara Sze Man Tang, Paul Kwong Hang Tam, Kenneth Kak Yuen Wong, Patrick Ho Yu Chung

**Affiliations:** 1 Department of Surgery, School of Clinical Medicine, Queen Mary Hospital, The University of Hong Kong, 102 Pokfulam Road, Hong Kong SAR, China; 2 Dr. Li Dak-Sum Research Centre, The University of Hong Kong, Hong Kong SAR, China; 3 Faculty of Medicine, Macau University of Science and Technology, Macau SAR, China

**Keywords:** Biliary atresia, Steroid, Liver fibrosis, Organoid, Genes

## Abstract

**Purpose:**

We performed animal and organoid study to evaluate the anti-fibrotic effect of steroid on biliary atresia (BA) and the underlying patho-mechanism.

**Methods:**

BA animal models were created by inoculation of mice on post-natal day 1 with rhesus rotavirus (RRV). They received either 20 µl phosphate-buffered saline (PBS) or steroid from day 21 to day 34. On day 34, their serum samples were collected for hormonal markers. Necrosis, fibrosis and CK 19 expression in the liver were evaluated. Liver organoids were developed and their morphology as well as bulk RNA sequencing data were analyzed.

**Results:**

Twenty-four mice developed BA features after RRV injection and were equally divided into steroid and PBS groups. On day 34, the weight gain of steroid group increased significantly than PBS group (*p* < 0.0001). All mice in the PBS group developed liver fibrosis but only one mouse in the steroid group did. Serum bilirubin and liver parenchymal enzymes were significantly lower in steroid group. The morphology of liver organoids were different between the two groups. A total of 6359 differentially expressed genes were found between steroid group and PBS group.

**Conclusion:**

Based on our findings obtained from RRV-induced BA animal and organoid models, steroid has the potential to mitigate liver fibrosis in BA.

## Introduction

Biliary atresia (BA) is an uncommon disorder characterized by progressive obliterative cholangiopathy of the intra- and extrahepatic bile ducts. This disorder always presents at neonatal and early infantile periods with persistent jaundice and the passage of acholic stool [1]. The disease incidence is around 1 in 10,000 to 16,700 among Caucasians [2–5]. However, it is more common in Asians and the incidence is up to 1 in 5000 to 8000. It is postulated that Rotavirus infection plays a key role in the pathogenesis that leads to biliary inflammation and fibrosis [6]. Kasai operation is the most widely accepted primary treatment for BA and liver transplant is reserved for late presenter or patient who have an unsuccessful Kasai operation [7, 8]. Unfortunately, up to 50% of patients develop liver fibrosis. Various adjuvant medications have been proposed to improve the success rate of Kasai operation and steroid is one of the most frequently prescribed medications. It was postulated that steroid is anti-inflammatory but its effect on fibrosis has not been examined [5, 9, 10].

Disease-related gene and signaling pathway therapy is a new way to treat rare diseases. According to published studies, it has been reported that damage to lipid metabolism pathway can lead to fibrosis, and the effect of lipid metabolism pathway in fibrosis has been confirmed [11–13]. Thus, the effect of steroids on the lipid metabolism pathway and liver fibrosis in BA is one of the research areas. We speculated that steroids may reduce the fibrotic symptoms of BA by regulating the lipid metabolism pathway process.

The introduction of animal model and organoid technique has played an important role in research of hepatobiliary diseases. BA animal models can be created by oral or intra-abdominal administration of Rhesus Rotavirus (RRV) [6]. RRV virus can lead to BA symptoms in mice including smaller body size, obstructive jaundice and liver fibrosis. Thus, RRV-positive mice are considered a suitable model to study interventions in BA. Recently, many studies on bile duct cell differentiation have been conducted at the level of liver organoids, “mini-organs” that produce cholangiocytes as well as hepatocytes in a 3-D level. BA organoid models have been successfully developed for the study of disease pathogenesis [14, 15]. Organoids from the RRV-infected mice exhibited aberrant morphologies, perturbed cholangiocytes development and expression profile, similar to that observed in BA patients’ liver organoids [16, 17].

In this study, we used cutting edge technology to explore specifically the anti-fibrotic effect of steroid in BA without prior Kasai operation. Animal and liver organoid models were the study tools to examine the phenotypical, histological, biochemical and molecular changes in BA treated with steroid. It was our objective to provide evidence from in-vitro study to bridge the gap towards clinical application.

## Materials and methods

### Generation of RRV-BA mouse model

Postnatal day 2 BALB/c mice (< 3 g) were injected with RRV (rhesus rotavirus; 20 µl of 1 × 106 pfu/ml RRV, MMU 18006, ATCC® VR-1739™) via peritoneal route. All animals were monitored daily after inoculation of RRV. The development of acholic stools, and jaundice on day 5 to 6 post-injection indicated a successful induction of BA. All the animal experiments were performed according to the animal ethics approvals (CULATR 5720–21) from the Faculty of Medicine, The University of Hong Kong, Hong Kong.

## Morphological and histological examination of RRV-BA mouse liver

Mouse neonates were sacrificeded on day 7, 14 and 34 post-RRV inoculation. Morphological examination of the liver and the gall bladder was performed to look for BA phenotypes. Liver tissues were fixed in 4% paraformaldehyde (w/v) in PBS for 48 h at 4 ℃, dehydrated in graded series of alcohol, and cleared in xylene before being embedded in paraffin. Sections (4 µm in thickness) were prepared and mounted onto TESPA-coated microscope glass. Sections were dewaxed in xylene, hydrated in a graded series of alcohol and finally in distilled water. Necrosis of liver sample was determined using haematoxylin and eosin (H&E) staining, dehydrated and mounted for microscopic analysis. For the detection of liver fibrosis, collagen was stained with Sirius Red according to manufacturer’s protocol (ab150681, Abcam).

## Steroid treatment of RRV-BA mice

Post-RRV-injected day 21 BA mice (*n* = 24) were equally divided into two groups. Mice that were allocated to steroid group received daily intraperitoneal injection of with prednisolone (Redipred, Aspen, Australia; 4 mg/kg body weight) from post-RRV day 21 to day 34. The other group of BA mice received daily intraperitoneal injection of same volume of PBS from post-RRV day 21 to day 34. All the mice in each group were weighted every day after birth until the day of killing.

## Liver function test

At the time of sacrifice, peripheral blood was collected from the inferior vena cava into Lithium heparin microvette (D-51588, Sarstedt) and centrifuges at 2000 g for 5 min at room temperature to collect the plasma. Plasma was sent to medical laboratory to determine plasma level of total bilirubin, alkaline phosphatase (ALP), alanine aminotransferase (ALT) and aspartate transaminase (AST).

## Immunohistochemistry

Paraffin sections (4 µm in thickness) of liver were dewaxed in xylene, hydrated in a graded series of alcohol and finally in distilled water. Antigen retrieval was performed by incubation in 10 mM sodium citrate buffer (pH 6.0) at 95 ℃ for 10 min. Endogenous peroxidase activity was blocked by 10-min incubation of slides in hydrogen peroxide methanol solution (30% H2O2 in methanol) followed by PBS wash (3 times, 5 min each). Sections were then incubated in blocking buffer (PBS with 0.1% Triton supplemented with 1% (w/v) Bovine Serum Albumin (USB Corporation, Cleveland, OH USA)) for 1 h at room temperature. After blocking, sections were incubated with anti-CK19 antibody (EP1580Y; abcam; ab52625; 1:1000 dilution) in PBS-T plus 1% BSA (w/v) for overnight at 4 ℃. The sections were washed in PBS-T and secondary body incubation and signal development were performed using EnVision Detection Systems Peroxidase/DAB, Rabbit/Mouse system according to manufacturer’s protocol (Dako). After washing in water, sections were counterstained with Haematoxylin, washed in water, incubated in 2% (W/V) NaHCO3 for 30 s before being washed in water. Sections were dehydrated in a graded series of alcohol, cleared in xylene and then mounted in DPX mountant (BDH). Images were taken with Nikon Eclipse E600 microscope mounted with Nikon Digital Camera DXM1200F.

## Liver organoids

### Liver organoids culturing

Liver tissues were minced in cold wash medium (Advanced DMEM/F12; 1% GlutaMAX; 1% FBS; 1% Penicillin/Streptomycin (P/S)) and transferred to a gentleMACS-C Tube (Miltenyi Biotec Inc. CA, USA). Wash medium was removed when the tissue pieces settled to the bottom of the tube. Samples were again washed with 5 ml of wash medium by pipetting up and down 10 times to remove red blood cells and fat, and the medium aspired when tissue pieces settled down. Minced liver tissues were digested with 5 ml of digestion medium (Multi Tissue Dissociation Kit 1; Miltenyi Biotec Inc. CA, USA) on gentleMACS™ Octo Dissociator (Miltenyi Biotec Inc. CA, USA) using program 37ºC-Multi-A-01. After digestion, cold wash medium (5 ml) was added to each tube, the mixture was filtered through a 70 µm strainer (Miltenyi #130-110-916) and then a 30 µm strainer (Miltenyi #130-110-915) before centrifugation (300 g; 10 min) to pellet the cells. The cell pellet was washed once with 5 ml of Advanced DMEM/F12, and then resuspended cells in 100 µl of MACS column buffer (PBS pH 7.2 + 0.5% BSA). CD326 (EpCAM) microbeads (10 µl microbeads/107 cells; #130-105-958; Miltenyi Biotec Inc. CA, USA) were added, followed by incubation of the mixture in the dark at 4 ℃ for 15 min. After incubation, 0.9 ml of column buffer was added and EpCAM-positive cells were sorted on an LS Column (Miltenyi #130-042-401) following the manufacturer’s protocol (Miltenyi Biotec Inc. CA, USA). The cells were pelleted (300 g; 10 min) and mixed with 35 µl of Matrigel (356,231; Corning Biocoat) and seeded per well of a prewarmed (37 ℃) 4-well plate (176,740; Nunc; Thermo Scientific). The cell-matrigel mixture was added to the well and plates (matrigel down) were incubated at 37 ℃ for 5 min initially; the plates were then incubated upside-down (matrigel up) at 37 ℃ for 5 min again to evenly distribute cells within the matrigel and to avoid accumulation of cells towards the bottom of the plates. After Matrigel had solidified, 500 µl organoid medium was added to each well. Organoid medium was based on Advanced DMEM/F12 (Invitrogen) supplemented with Penicillin/Streptomycin (Invitrogen), GlutaMax (Invitrogen), 25 mM HEPES (Invitrogen), 1% N2 (GIBCO), 1% B27 (GIBCO), 1.25 mM Acetylcysteine (Sigma), 10 nM gastrin (G9145; Sigma), 50 ng/ml EGF (PMG8043; PeproTech), 100 ng/ml FGF10 (100–26-25UG; PeproTech), 25 ng/ml HGF (100–39-10UG; PeproTech), 10 mM Nicotinamide (Sigma), 5 µM A83.01 (Tocris), 10 µM Forskolin (Tocris), 500 ng/ml R-Spondin 1 (7150-RS-025; R&D), 100 ng/ml Noggin (250-38-20UG; Peprotech), 100 ng/ml Wnt3a (1324-WN-010; R&D) and 250 ng/ml Amphotericin B (#15290018; GIBCO). For the first 6 days of culture, 10 µM ROCK inhibitor Y-27632 (Tocris) was added. Medium was changed once every 2 days. Passage zero (P0) primary organoids of day 34 control and prednisolone-treated and untreated BA liver were used for all the experiments.

## Bulk RNA sequencing and transcriptomic analysis of organoids

Organoids were retrieved from Matrigel to individual tubes (one organoid per tube and eight organoids for each group) for RNA sequencing. Organoid lysis, total RNA preparation, reverse transcription, amplification and library construction were performed using single cell RNA-seq technology (Smart-seq2.0) with minor modifications for generating bulk RNA transcriptomics to get good depth in the transcriptome analysis as previously reported [15]. Library construction was performed using Nextera XT Kit following the manufacturer’s protocol. Libraries were pooled and sequenced by pair ends of 100 base pairs (PE100) on an illumine HiSeq 2500 System. Transcriptomic analysis was performed following the pipeline as shown on Fig. 4.

## Statistical analysis

Data from separate quantitative analyses were compiled and analyzed between groups. The Shapiro–Wilk test was used to check for normality prior to performing the comparison using Student’s 2-tailed *t*-test, and *p* < 0.05 was regarded as statistically significant.

## Results

### Number of animal models created

Fifty mice received RRV injection and six mice were used as control without RRV injection. Among 50 mice with RRV injection, 24 developed BA symptoms and they were divided into steroid (*n* = 12) and PBS (*n* = 12).

## Steroid treatment improves the growth and jaundice clearance of RRV-induced BA mice

**Fig. 1:**
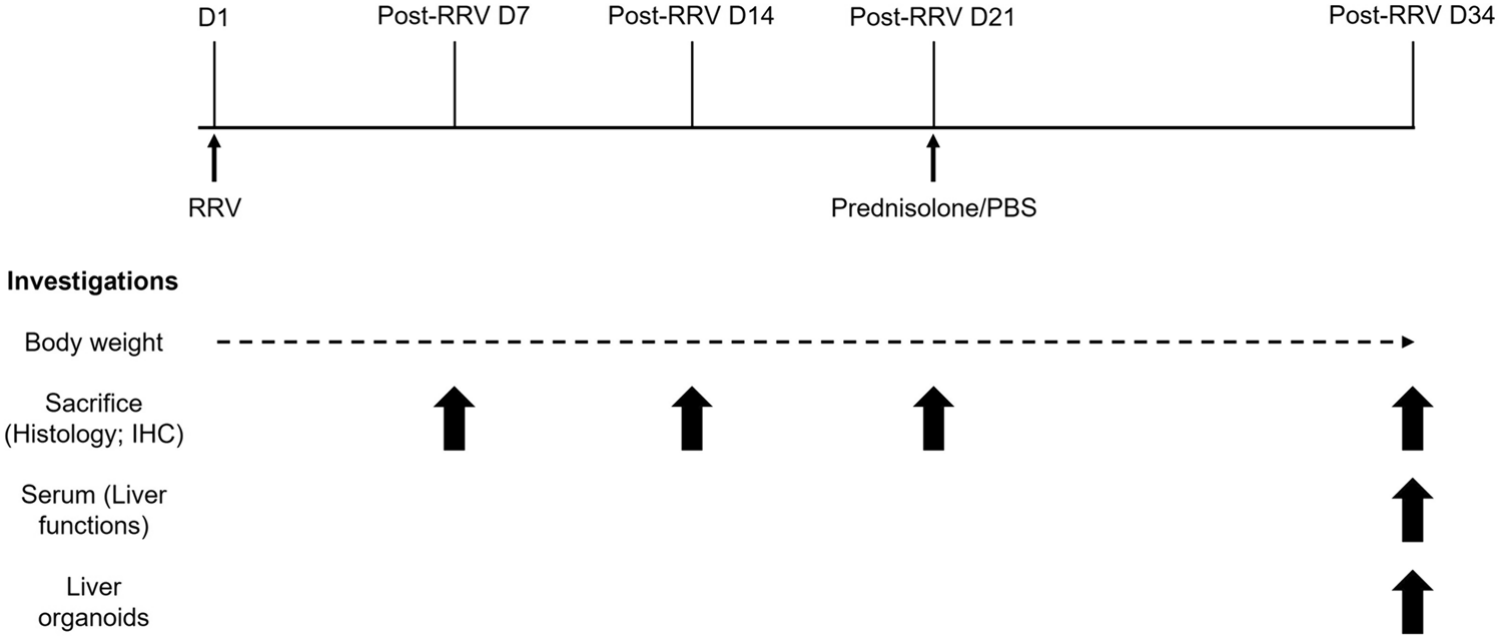
Experimental plan to investigate the effects of prednisolone in disease progression in RRV-induced BA mice. Dotted line indicates daily body weight measurement of mice of different groups. The post-RRV day at which mice were sacrficed for different investigations were indicated by upward arrows

**Fig. 2:**
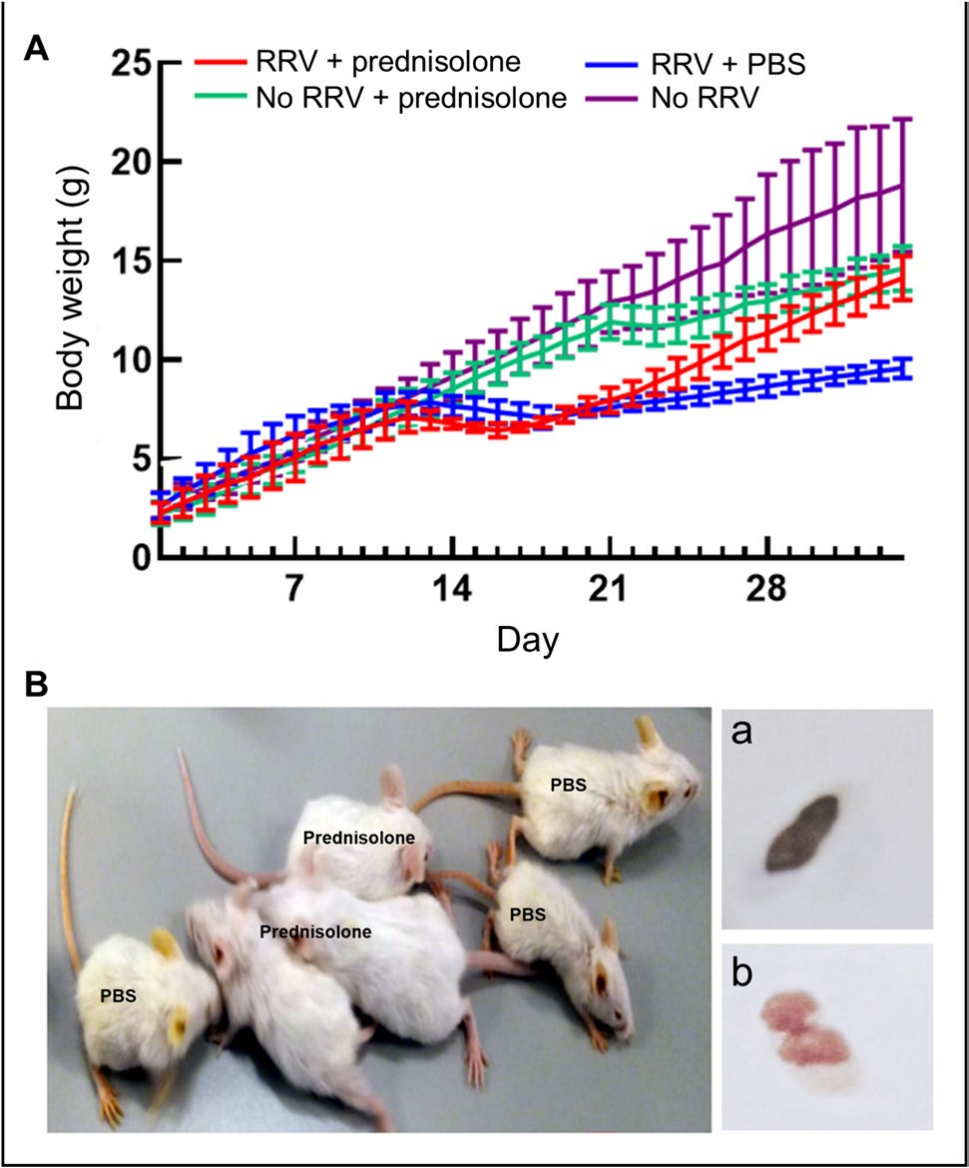
RRV-induced BA mice showed better growth and clearance of jaundice after prednisolone treatment. (**A**) Daily body weight of control mice (No RRV; *n* = 3), prednisolone only mice (No RRV + prednisolone (*n* = 3), prednisolone-treated RRV-injected mice (RRV + prednisolone; *n* = 9) and PBS-treated RRV-injected mice (RRV + PBS; *n* = 9). (**B**) Photos of RRV-injected mice in PBS and prednisolone treatment group at post-RRV day 34. Photos of stools of post-RRV day 34 PBS-treated RRV-injected mice (**a**) and post-RRV day 34 prednisolone-treated RRV-injected mice (**b**)

We established RRV-induced BA mouse model and use this model to investigate if steroid treatment could ameliorate disease progression with experimental setup depicted as shown on Fig. 1. To monitor the disease progress in BA mice before and after treatment, we recorded the daily body weight of mice of each group from day 1 to day 34. Body weight of control mice (*n* = 3; No RRV: without RRV injection, PBS injection from day 14 to day 34) increased lineally from 2.7 ± 0.6 g (mean ± SD) on day 1 to 18.8 ± 3.4 g (mean ± SD) on day 34 (purple line, Fig. 2A). RRV-injected mice showed a body weight increase from day 1 to day 12 (red and blue lines, Fig. 2A), and their body weight was not significantly different from that of control mice. However, body weight of RRV-injected mice (*n* = 18) started to deviate from control mice from day 12, and their body weight was consistently lower than that of the controls (Fig. 2A).

Staring from day 14, half of the RRV-injected mice received daily prednisolone and the other half received daily PBS. In prednisolone treatment group, body weights of mice (*n* = 9) did not change much in the first 2 days after treatment, but their body weights started to increase lineally from 6.6 ± 0.2 g (mean ± SD) at day 17 to 14.1 ± 1.1 g (mean ± SD) at day 34. RRV-injected mice in PBS group (n = 9) showed a drop of body weight from 7.7 ± 0.5 g (mean ± SD) at day 14 to 7.4 ± 0.3 g (mean ± SD) at day 20; their body weights increased slowly from day 21 onwards, but they were significantly lighter than the prednisolone-treated RRV-injected mice of the respective days. At the day of killing at day 34, control mice have the highest body weight, followed by prednisolone-treated RRV-injected mice, and then by PBS-treated RRV-injected mice (control vs prednisolone vs PBS; 18.8 ± 3.4 g (mean ± SD) vs 14.1 ± 1.1 g (mean ± SD) vs 9.5 ± 0.5 g (mean ± SD); p < 0.05) (Fig. 2A). The body weight of only prednisolone-treated mice (No RRV + prednisolone; green line) showed similar trend as the control mice from day 1 to day 21 (Fig. 2A). Increase of body weight of the only prednisolone-treated mice slowed down from day 21 onwards, their body weights were lower than the control mice, but they are similar to those of prednisolone-treated RRV-injected mice at day 34.

As shown in Fig. 2B, day 34 prednisolone-treated RRV-injected were larger, did not show signs of jaundice with black colored stool (Fig. 2B and Ba). In contrast, PBS-treated RRV-injected were smaller than their age-matched counterparts, showed obvious signs of jaundice with pale yellow colored stool (Fig. 2B and Bb).

**Fig. 3:**
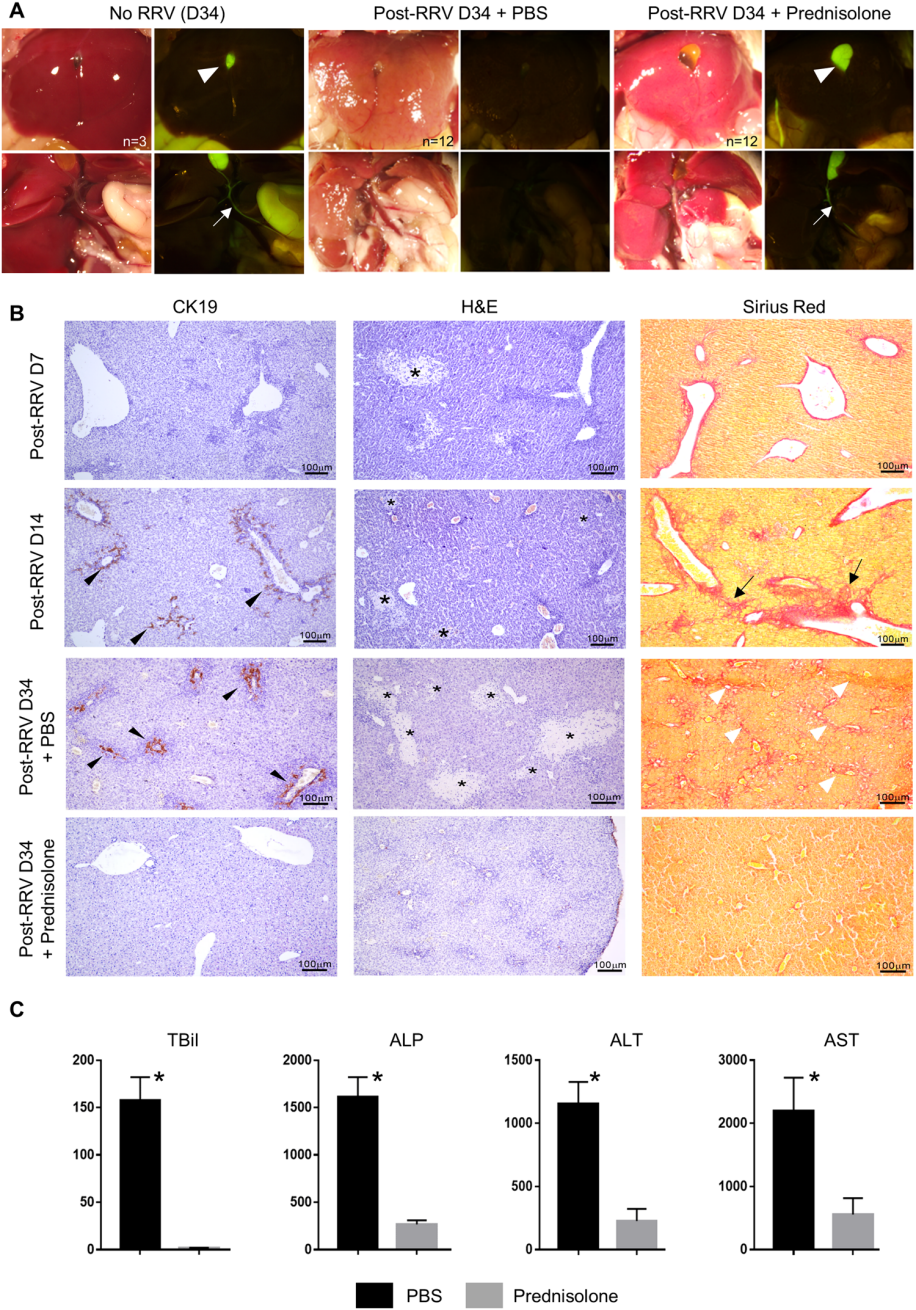
Prednisolone treatment mitigates BA disease progression in RRV-induced BA mice. (**A**) Representative photos of livers of mice of different groups at post-RRV day 34. (**B**) Representative photos of CK19 immuno-stained, H&E stained and Sirius Red-stained liver sections of mice from post-RRV day 7 and day 14 mice, PBS and prednisolone-treated RRV-injected mice at post-RRV day 34. (**C**) Serum bilirubin and liver enzyme level of PBS and prednisolone-treated RRV-injected mice at post-RRV day 34 (*n* = 9 in each group). “*” indicates *p* < 0.0001

Control mice, prednisolone-treated and PBS-treated RRV-injected mice were sacrificed on post-RRV day 34 for morphological examination of the liver and gall bladder. As shown in Fig. 3, for the mice that did not receive RRV injection (No RRV), the liver was bright red with a smooth surface, and the gall bladder was distended. In contrast, the liver was pale and nodular while the gall bladder was poorly-shaped among PBS-treated RRV-injected mice (post-RRV D34 + PBS). Steroid treatment improved the growth and jaundice clearance of RRV-injected BA mice. However, in prednisolone-treated RRV-injected mice (post-RRV D34 + prednisolone), the liver was less pale and the surface was smooth.  There wereno white nodulese observed and the gall bladder was well formed. Under green fluorescence illumination, the gall bladder (arrowhead) and the extrahepatic bile duct (arrow) were clearly seen (as shown with green fluorescence) in control and prednisolone-treated RRV-injected mice. In PBS-treated RRV-injected mice, the gall bladder was atretic and the extrahepatic bile duct was not patent.

Next, we performed histology and Sirius Red staining of the liver sections of mice of different groups to further assess the condition of the livers of these mice. H&E staining clearly revealed areas of necrosis in the liver of post-injection day 7 and day 14 RRV-injected BA mouse (*; Fig. 3B). Areas of necrosis (*) were detected in the livers of PBS-treated RRV-injected mice, whereas necrosis was not found in the livers of prednisolone-treated RRV-injected mice (Fig. 3B). Sirius Red staining revealed fibrosis at the portal areas of the post-RRV day 14 mouse livers (arrow; Fig. 3B), but no fibrosis was detected in post-RRV day 7 mouse livers. Fibrosis was revealed at the portal areas and the interlobular regions of the livers of PBS-treated RRV-injected mice (arrow; Fig. 3B), but no fibrosis was detected in the livers of prednisolone-treated RRV-injected mice. Ductular proliferation (arrow) was detected in the post-RRV day 14 mouse livers and the livers of PBS-treated RRV-injected mice by CK19 immunostaining at the portal areas (Fig. 3B). No ductular proliferation was detected in the livers of prednisolone-treated RRV-injected mice.

Plasma levels of total bilirubin (TBil), alkaline phosphatase (ALP), alanine aminotransferase (ALT) and aspartate aminotransferase (AST) were markedly elevated in post-RRV day 34 PBS-treated mice and were statistical significantly higher (*p* < 0.0001) than that of prednisolone-treated RRV-injected mice (Fig. 3C).

## Defective lipid metabolism in RRV-induced BA liver tissue-derived organoids

**Fig. 4:**
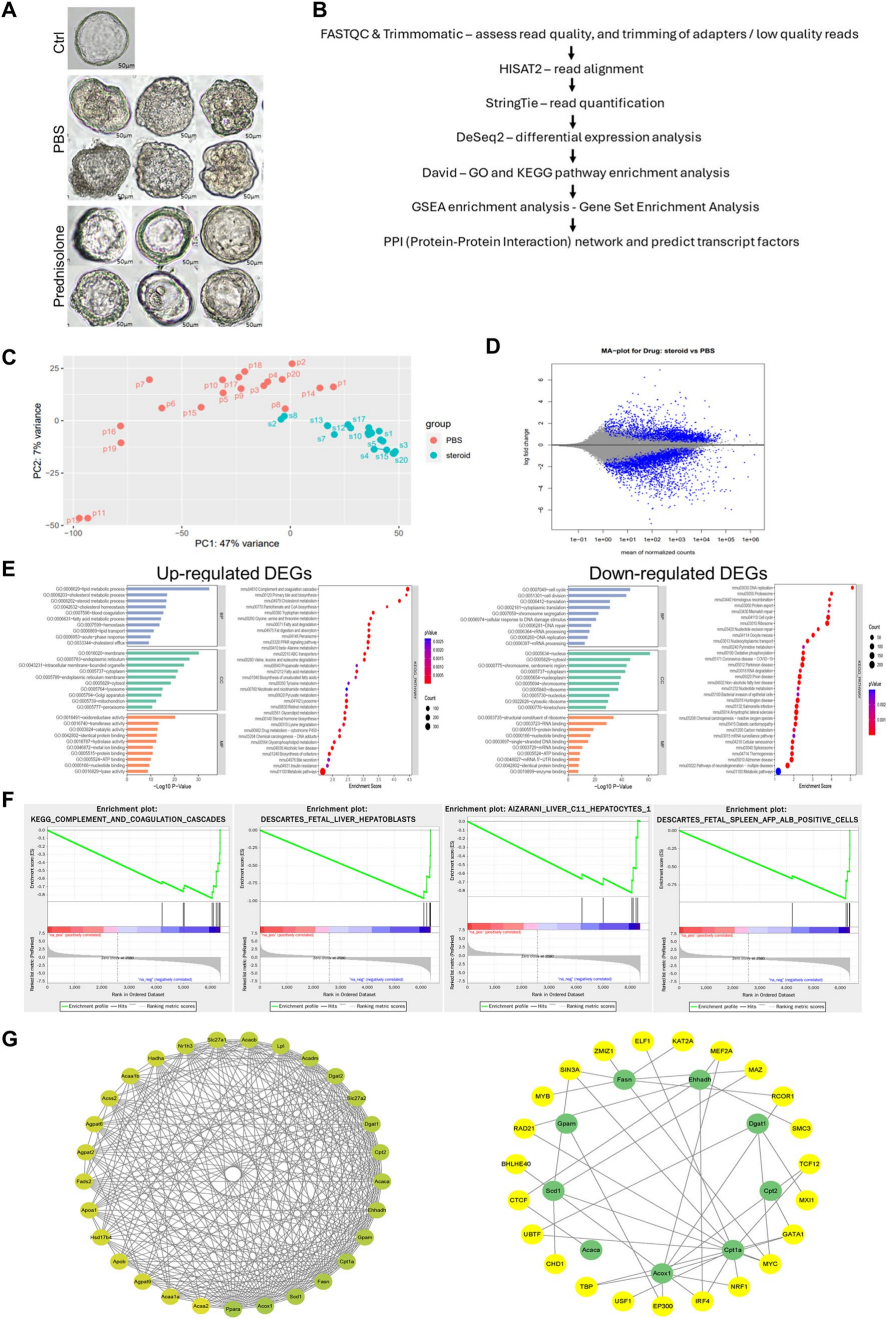
RNA sequencing and bioinformatics analysis of liver tissue-derived organoids of prednisolone and PBS-treated RRV-induced BA mice. (**A**) Photos of BA liver tissue-derived organoids of control mice (Ctrl), prednisolone-treated RRV-injected mice (prednisolone) and PBS-treated RRV-injected mice (PBS). (**B**) Bioinformatic analysis pipeline. (**C**) Plot of principal component analysis of transcriptomes of liver tissue-derived organoids of prednisolone-treated RRV-injected mice (steroid; blue) and PBS-treated RRV-injected mice (PBS; red). (**D**) MD plot showing the log-fold change and average abundance of each gene (steroid vs PBS). (**E**) GO and KEGG pathway enrichment analysis of up-regulated DEGs. (**F**) GO and KEGG pathway enrichment analysis of down-regulated DEGs. (**G**) Protein–protein interaction (PPI) analysis and transcription factors prediction of DEGs

Organoids were generated from liver tissues of control, post-RRV day 34 prednisolone and PBS-treated mice. Normal liver tissue-derived organoids were large, well expanded with a single cystic structure and a single layer of epithelial cells (Ctrl; Fig. 4A). In the prednisolone treatment group, over 83% (20/24) of organoids were large, well expanded with a single cystic structure and a single layer of epithelial cells (Fig. 4A). In contrast, over 90% (47/52) of organoids in PBS treatment group were poorly expanded, with some organoids showing multi-vacuole feature (*). Bulk RNA sequencing and bioinformatic analysis were performed to investigate the differential transcriptomes of organoids of prednisolone and PBS treatment groups using the pipeline as shown in Fig. 4B. Principal component analysis (PCA) showed a separate clustering of organoids of prednisolone and PBS treatment groups (Fig. 4C). We identified a total of 6359 differentially expressed genes (DEGs; 2580 up-regulated genes and 3379 down-regulated genes) between organoids of prednisolone and PBS treatment groups (Fig. 4D). GO and KEGG functional enrichment analysis showed that: down-regulated genes mainly affect metabolic pathways (especially lipid metabolism, cholesterol metabolism, steroid metabolism), followed by complement and coagulation cascades and PPAR signaling pathways, which associated with fatty acid transporters mostly; up-regulated genes are related to ribosome and cell cycle process (Fig. 4E). GSEA KEGG pathway enrichment analysis showed that all the 6359 DEGs were mostly related to complement and coagulation cascades. GSEA enrichment analysis of all cell type signature gene sets showed that fetal liver hepatoblast, fetal spleen AFP ALB-positive cells and hepatocytes are the main cell types enriched for all the 6359 DEGs (Fig. 4F). Next, we performed protein–protein interaction network analysis to identify hub genes in lipid metabolism process and predict their transcript factors (Fig. 4G). The first 10 hub genes in lipid metabolism process were identified to be Acos1 (Acyl-CoA Oxidase1), Ppara (Peroxisome Proliferator Activated Receptor Alpha), Scd1 (stearoyl-CoA desaturase-1), Fasn (Fatty Acid Synthase), Cpt1a (Carnitine Palmitoyl-Transferase1A), Gpam (Glycerol-3-Phosphate Acyltransferase, Mitochondrial), Cpt2 (Carnitine palmitoyltransferase 2), Acaca (Acetyl-CoA carboxylase alpha), Dgat1 (Diacylglycerol O-Acyltransferase 1) and Ehhadh (Enoyl-CoA Hydratase And 3-Hydroxyacyl CoA Dehydrogenase) (shown in green dot), respectively, with predicted transcript factors on the outside of the circle (shown in yellow dot).

## Discussion

Previous clinical studies have provided evidence that post-Kasai usage of steroid improves biliary drainage possibly by promoting canalicular electrolyte transport and stimulating bile salt-independent bile flow. However, the underlying molecular mechanism behind the action of steroid remains undetermined. Furthermore, previous studies focused on steroid usage after Kasai operation but its effect as a neo-adjuvant to alleviate liver fibrosis before Kasai operation is unknown.

Our findings have provided preliminary data regarding the effect of steroid on BA mice without Kasai operation. We noticed that among RRV infected mice, steroid was able to reduce cholestatic symptoms, increase weight gain, reverse undesirable morphology, and improve liver biochemistry. Moreover, steroid played an important role in modulating CK19 expression, liver necrosis and fibrosis. These observations were also confirmed by the molecular details in liver organoids developed from the mouse models. To test our hypothesis that steroid could mitigate liver fibrosis via its effects on lipid metabolism pathway, we performed transcriptomic analysis. Interestingly, we were able to identify differentially expressed genes (DEGs) in PBS treatment group and steroid treatment group. All the down-regulated differential genes codirected to the lipid metabolism process pathway. This finding has provided data to support our hypothesis. Indeed, some recently published reports also described the relationship between lipid metabolism and liver fibrosis [18, 19]. For example, if fatty acid synthetase is defective, mitochondrial fusion proteins can aggravate pulmonary fibrosis by inhibiting lipid synthesis. Therefore, it was believed that mitochondrial fusion is closely related to lipid metabolism and the progression of fibrosis [12]. Another example is the discovery of lipid toxicity as the main cause of renal fibrosis. The symptoms of renal fibrosis can be alleviated by regulating the fatty acid oxidation process in kidney cells [20]. Fibrosis represents anomalous precipitation of extracellular matrix that leads to malfunction of organs, disease and demise. Preventing or reversing fibrosis has been challenging and anti-fibrotic agents currently available are not always effective. Together with our findings and other reports that metabolic dysregulation could lead to fibrosis, drugs that target these pathways may become a new avenue of anti-fibrotic treatment.

In conclusion, our study has provided convincing evidence that steroid could ameliorate liver fibrosis and other symptoms in BA mice. Notably, this novel finding was obtained from animal without Kasai operation and hence representing neo-adjuvant usage. Based on transcriptomic analysis, the mechanism was believed to be related to lipid metabolism. Future works that focus on potential medications that regulate lipid metabolism and the subsequent fibrosis are recommended with an ultimate aim to improve the prognosis of BA.

## Data Availability

No datasets were generated or analyzed during the current study.
